# Manual of Orthopedic Surgery, Being a Dissertation Which Obtained the Boylston Prize for 1844, on the Following Question: “in What Cases and to What Extent Is the Division of Muscles, Tendons, or Other Parts Proper for the Relief of Deformity or Lameness?”

**Published:** 1845-07

**Authors:** 


*Manual of Orthopedic Surgery, being a Dissertation which obtained the Boylston prize for* 1844, *on the following question: " In what cases and to what extent is the division of Muscles, Tendons, or other parts proper for the relief of deformity or lameness ?"* By JACOB BIGELOW, M. D. 8vo., pages 211, with several plates. William D. Ticknor & Co., Boston ; 1845.

In this work the author has given an account of the various operations, instruments and means employed or proposed, in "*Strabismus, Stammering, Tenotomy, Club-foot, Torticollis, False Anchylosis of the Knee-joint, Ricketty Knees, Permanent Flexion of the Hip-joint, Anchylosis, Lateral Curvative of the Spine, Contraction of the Hand and Fingers, Congenital Dislocations, Recent aud Chronic Dislocations, Section of Muscles in Locked-jaw, Subcutaneous Section of the Orbicular Muscles,*" and an *Appendix*, containing directions for taking casts in plaster.

This is a neat little book, both as regards its literary and mechanical execution, and we are persuaded it will be received by the profession as a concise and useful manual on the subjects it embraces. In the hasty glance we have been able to give it, however, we have been particularly struck with two circumstances ; viz. the singular avoidance of the points contained in the question submitted for the prize, and the almost total neglect of every thing *American*, relating to the subjects on which the book is written. The latter is the more remarkable, because not only have many of the operations treated of been performed by several of our own surgeons, of which accounts have appeared in the Journals, but details of many of them are contained in Reese's edition of Cooper's Dictionary, and likewise in the appendix to Mott's edition of Velpeau—works which could hardly have escaped the attention of so well read a physician as Dr. Bigelow, particularly when preparing a work on an interesting branch of modern Surgery.

